# Evaluating a surveillance system: live-bird market surveillance for highly pathogenic avian influenza, a case study

**DOI:** 10.11694/pamj.supp.2014.18.1.4188

**Published:** 2014-07-21

**Authors:** Ndadilnasiya Endie Waziri, Patrick Nguku, Adebola Olayinka, Ike Ajayi, Junaidu Kabir, Emmanuel Okolocha, Tesfai Tseggai, Tony Joannis, Phillip Okewole, Peterside Kumbish, Mohammed Ahmed, Lami Lombin, Peter Nsubuga

**Affiliations:** 1 Nigeria Field Epidemiology and Laboratory Training Program, Nigeria; 2 Department of Epidemiology, Medical Statistics and Environmental Health, University of Ibadan, Nigeria; 3 Department of Veterinary Public Health, Ahmadu Bello University, Zaria, Nigeria; 4 ECTAD Unit, Food and Agriculture Organization, Bangladesh; 5 National Veterinary Research Institute, Vom, Nigeria; 6 Global Public Health Solutions, Decatur GA, USA

**Keywords:** Live bird market, highly pathogenic avian influenza, surveillance, Nigeria

## Abstract

**Introduction:**

Highly pathogenic avian influenza H5N1 was first reported in poultry in Nigeria in February 2006. The only human case that occurred was linked to contact with poultry in a live bird market (LBM). LBM surveillance was instituted to assess the degree of threat of human exposure to H5N1. The key indicator was detection of H5N1 in LBMs. We evaluated the surveillance system to assess its operations and attributes.

**Methods:**

We used the US Centers for Disease Control and Prevention (CDC) updated guidelines for evaluating public health surveillance systems. We reviewed and analyzed passive surveillance data for HPAI (January 2006-March 2009) from the Avian Influenza National Reference Laboratory, and live bird market surveillance data from the Food and Agriculture Organization of the United Nations, Nigeria. We interviewed key stakeholders and reviewed reports of live bird market surveillance to obtain additional information on the operations of the system. We assessed the key system attributes.

**Results:**

A total of 299 cases occurred in 25 (72%) states and the Federal Capital Territory (FCT). The system detected HPAI H5N1 virus in 7 (9.5%) LBMs; 2 (29%) of which were from 2 (18.2%) states with no previous case. A total of 17,852 (91.5%) of samples arrived at the laboratory within 24 hours but laboratory analysis took over 7 days. The sensitivity and positive predictive value (PPV) were 15.4% and 66.7% respectively.

**Conclusion:**

The system is useful, flexible, complex and not timely, but appears to be meeting its objectives. The isolation of HPAI H5N1 virus in some of these markets is an indication that the markets are possible reservoirs of the virus in Nigeria. We recommend that the Federal Government of Nigeria should dedicate more funds for surveillance for HPAI as this will aid early warning and reduce the risk of a pandemic.

## Introduction

Public health surveillance is the ongoing, systematic collection, analysis, interpretation and dissemination of data regarding a health-related event for use in public health to reduce morbidity and mortality and to improve health [1, 2]. Whether a surveillance system is passive or active, periodic evaluation is necessary in order to ensure problems of public health importance are being monitored efficiently and effectively and the evaluation should include recommendations for improving its quality, efficiency and usefulness [3]. Evaluation of a surveillance system focuses on how well the system operates to meets its purpose and objectives, and should involve an assessment of system attributes such as simplicity, flexibility, data quality, acceptability, sensitivity, predictive value positive, representativeness, timeliness, and stability. It is important to note that because public health surveillance systems vary in methods, scope, purpose and objectives, attributes that are important to one system might be less important to another. An outbreak of highly pathogenic avian influenza (HPAI) was first reported in Nigeria in February, 2006. Despite control measures including depopulation of affected farms and farms within 3 km radius, movement control and improved bio-security measures implemented to stop the spread of the disease, the virus rapidly spread to 25 states including the Federal Capital Territory (FCT) [4, 5] causing loss of over 1 million poultry. Although the World Health Organization (WHO) reported more than 628 confirmed human cases of avian influenza A (H5N1) globally, approximately two thirds of whom died [6], the first and only confirmed human H5N1 infection in Nigeria was reported in February 2007 [4] which was traced back to contact with infected poultry in a live bird market (LBM). LBMs are said to be the most important mixing point for all birds and have been implicated in the spread of H5N1 HPAI. While birds from large and small-scale commercial sectors and scavenging poultry mix in these markets, traders and other intermediaries also serve as vehicles for HPAI transmission. Live bird market surveillance (LBMS) for HPAI was instituted by the Nigeria government in collaboration with the Food and Agriculture Organization (FAO) to improve the understanding of the role of LBMs in the epidemiology of HPAI in Nigeria and the degree of threat of human exposure to HPAI virus. We evaluated the LBMS for HPAI to assess its operations, evaluate its key system's attributes and assess whether the system is meeting its objectives.

## Methods

### Data collection and review

We obtained monthly passive surveillance data for HPAI (January 2006-March 2009) from the Avian Influenza National Reference Laboratory, and live bird market surveillance data from the FAO, Nigeria. The data were compiled and reviewed for errors. A case of HPAI was one with a positive laboratory result by agar gel immune diffusion test. We analyzed the data and compared monthly trends of the disease. We performed descriptive epidemiology to summarize the data in person, place and time. A state with a positive laboratory test either from the passive surveillance or the live bird market surveillance was considered infected. We analyzed data from the surveillance for both the infected and non-infected states.

### System evaluation

We used the US Centers for Disease Control and Prevention (CDC) updated guidelines for evaluating public health surveillance systems [3]. We assessed the operations of the system and the system's attributes. We interviewed key stakeholders and reviewed reports of live bird market surveillance to obtain additional information on the operations of the system. We reviewed data collection tools and raw data from two states; one each from an infected and non-infected state to check for consistency in data and errors in data collection and entry, and the ease of use of the tools. Laboratory results in the reports were compared with the results in the laboratory. We assessed the key system attributes (simplicity, flexibility, acceptability, data quality, stability, timelines, sensitivity and predictive value positive).

## Results

### Operation of the live bird market surveillance for highly pathogenic avian influenza system

**Figure 1:**
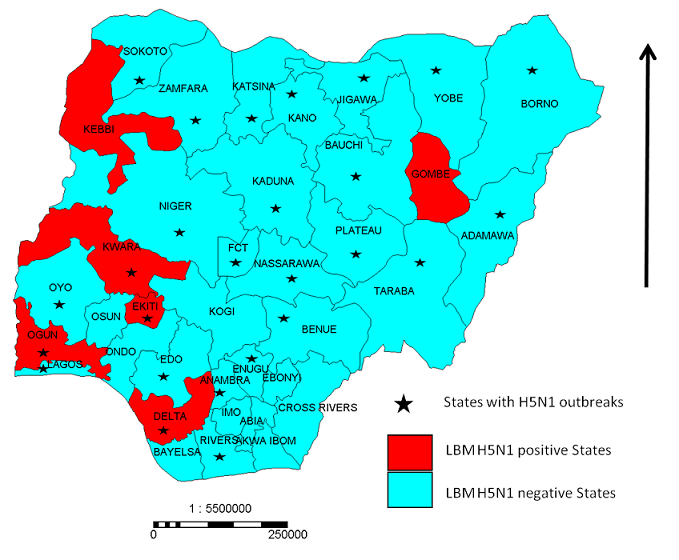
Map of Nigeria showing LBM H5N1 positive states and states with H5N1 outbreaks

Funding was provided by United States Agency for International Development (USAID) and the European Union (EU) with FAO acting as implementing partner. The indicators of the system are the detection of HPAI virus in LBMs, increased capacity of government to respond to HPAI occurrence in these markets, improved hygienic/biosecurity practices in LBMs and reduced risk of human exposure to HPAI virus. It has 13 stakeholders comprising international agencies, federal, state and local government agencies, private organizations and vocational associations and was operated in 2 phases. Phase I was instituted in the 26 states (including the Federal Capital Territory) that recorded cases during the H5N1 avian influenza outbreak in Nigeria that occurred between February 2006 to November 2008, while Phase II was done in the 11 states with no poultry outbreak of H5N1 avian influenza (Figure 1). In both activities, two markets per state comprising of one major market in the capital of each of the state which serves as the receiving depot for in-coming birds and distribution outlet to other parts of the state and a second market outside the state capital chosen based on size, number as well as types of birds sold. Sampling was done in each market at forth nightly intervals.

This was to take care of the assumption that all birds in the market at a particular time would be sold within two weeks. A total of 74 LBMs were used. A structured questionnaire was used to interview the traders. The questionnaire was designed to obtain information on name of market, demographics of traders, types and number of birds at point of sampling, sources of birds, type of housing used for birds, days of market operation, type of biosecurity measure practiced and days of resting of markets. Whole carcasses, as well as cloacae and tracheal swabs from both sick and apparently healthy birds were collected from each interviewed trader. The samples were sent to the National Veterinary Research Institute, Vom for laboratory analysis. In the laboratory, specimens were pooled weekly and tested for H5N1 by Agar Gel Immuno-diffusion, virus isolation, and molecular techniques.

### Passive surveillance

From the passive surveillance, of 1,675 suspected cases, 299 (17.9%) were confirmed from 25 (72%) states and FCT. There were 129 (43.1%) cases in 2006, 168 (56.2%) in 2007 and 2 (0.7) in 2008. In 2006, only 20 (54%) states including FCT had cases of HPAI, while in 2007, 4 (20%) of the 20 states with cases in 2006, did not record any case. Six (16%) states that did not have any case in 2006 had in 2007. Only two (5.4%) states recorded cases in 2006, 2007 and 2008, while four (10.8%) had cases in 2006 only. In 2006 the highest 46 (35.7%) of the cases were recorded in February while in 2007, 52 (31.0%) and 53 (31.6%) of the cases occurred in January and February respectively. In 2008, the two cases occurred in August. As at April 2013, no cases have occurred since November 2008.

### Usefulness of the System

The system generated data of LBMs operations and bio-security practices in the markets. It highlighted the need for Newcastle disease (ND) surveillance in Nigeria, as samples were collected in duplicates for future ND virus test. The system also detected HPAI H5N1 virus in 7 (9.5%) LBMs, 2 (29%) of which were from 2 (18.2%) states with no previous case or outbreak of H5N1.

### System Attributes

**Simplicity:** Although forms and questionnaires used in LBMS are easy to fill and the questions direct, diagnosis is strictly laboratory-based thus requiring specialized trainings for personnel. The test carried out was polymerase chain reaction, agar gel immuno-diffusion, and virus isolation. These tests are expensive, highly specialized and need trained personnel to carry out. Case definition for inclusion as HPAI also requires a positive result from one of the test procedures. No rapid test was used on the field and samples were shipped to the national reference laboratory for diagnosis.

**Flexibility:** The system was first designed for states with HPAI and it was later adapted for the states without HPAI. It also incorporated surveillance for Newcastle disease viruses.

**Data quality:** Questionnaires were administered by trained field officers. This explains why only 21 (1.6%) of questionnaires had missing values. Data collected were analyzed by trained veterinary epidemiologists.

**Acceptability:** Difficulties were encountered initially but with advocacy and sensitization visits to the markets and local authorities, there was full participation of all stakeholders. There was also a good public-private partnership as each team had a private veterinarian.

**Timeliness:** In phase I, 13,182 (95%) samples arrived the laboratory in good condition while in phase II, all samples reached the lab in good condition and within 24-72 hours of collection. No rapid test was used in the diagnostic procedures and laboratory analysis took over 7 days from time of arrival of specimens at the laboratory.

**Sensitivity and positive predictive value:** The proportion of states with HPAI detected by the surveillance system compared to the number of states with actual outbreaks of HPAI, which is the sensitivity of the system was 15.4% while the proportion of states with reported cases that actually had the virus in LBMs, which is the positive predictive value was 66.7%.

**Stability:** LBMS has dedicated personnel and an operational structure but it has no structured funding procedures as it is mainly donor driven.

## Discussion

The LBM surveillance system has helped in the generation of data on LBMs, their operations, biosecurity practices and the role they play in the epidemiology of H5N1 in Nigeria. It also identified markets that could be re-positioned due to the possible public health risk they pose. It has also highlighted the need for Newcastle Disease surveillance in Nigeria as an integral part of Avian Influenza control. The avian influenza epidemic in Nigeria started in January 2006 and seems to have come to an end in October 2008 with 25 states and the FCT recording at least a case each and 2007 recording the highest number of cases. After nine months without any positive case, the surveillance system picked two positive cases from two previously uninfected states. As at December 2012, no additional case has been reported. This could be attributed to the control measures of depopulation and decontamination of infected farms instituted by the Government. More cases were recorded in January and February of 2006 and 2007. These are the coldest time of the year in Nigeria and influenza viruses have been found to thrive more during cold seasons [7, 8].

We found the live bird market surveillance for highly pathogenic avian influenza to be useful with good data quality. The system was able to detect H5N1 virus in poultry in two markets that are situated in states that had recorded no case of HPAI during the epidemic. These cases would have otherwise been missed and the birds sold out putting handlers and consumers at risk of infection. These findings further support that LBMs are a potential source of H5N1. HPAI viruses have been isolated from live birds and poultry meat sold at markets in Thailand [9, 10]. Even though the system was first designed for infected states and only avian influenza, uninfected states were later included and also surveillance for New Castle disease done alongside avian influenza. Of note is the quality of personnel in the system. This was reflected in the quality of data collected by the system.

We found the system to be complex, not timely and not stable. We judged the system as complex based on the fact that every case had to be lab confirmed and the tests carried out need specialized training. The delay in the laboratory analysis and results is a pointer that positive birds could have been sold to the public for consumption.

## Conclusion

The System is useful, flexible and, has good data quality; however it is not timely and not stable, but appears to be meeting its objectives. It is important to note that even though the number of LBMs studied might not be representative the isolation of HPAI H5N1 virus in some of these markets is an indication that the markets are possible reservoirs of the virus in Nigeria and the delay in Lab analysis could result in selling of infected birds. Based on our findings, we recommended that the Federal Government of Nigeria should dedicate more funds for surveillance for HPAI as this will aid early warning and reduce the risk of a pandemic. Also, a rapid field test with robust sensitivity and specificity should be incorporated in the diagnostics procedures as this will reduce the time interval between sample collection and receipt of laboratory result. Finally, the isolation of HPAI H5N1 virus in LBMs is an indication that these markets are possible reservoirs of the virus in Nigeria therefore surveillance in more LBMs should be done. Surveillance is now being done in more markets to ensure that apparently healthy birds are not serving as a reservoir for the virus and also to help rapid response in case of an outbreak.
